# Cerebral blood velocity responses during and after sprint interval exercise in healthy young adults

**DOI:** 10.14814/phy2.70901

**Published:** 2026-05-01

**Authors:** E. Scott, E. Langson‐Justice, O. Smail, A. B. Lester, R. Oliveira, C. J. A. Pugh, M. E. Weston, B. O. Bond

**Affiliations:** ^1^ The Vascular Research Group, Public Health and Sport Sciences University of Exeter Exeter UK; ^2^ Federal University of Rio Grande Do Norte Natal Brazil; ^3^ Centre for Cardiovascular Research, Innovation and Development Cardiff Metropolitan University Cardiff UK; ^4^ THRIVE Laboratory, Department of Physiology, School of Medicine Trinity College Dublin Dublin Ireland

**Keywords:** autoregulation, cerebrovascular, pressure‐flow relationship, repeated sprints, sprint interval exercise

## Abstract

This study investigated the middle (MCAv) and posterior (PCAv) cerebral artery blood velocity and end‐tidal carbon dioxide (P_ET_CO_2_) responses during sprint interval exercise (SIE). We also examined the relationships between MCAv, PCAv and blood pressure during the 60 min post SIE. Fourteen healthy adults completed four 30 s maximal cycle sprints, each separated by 4 min of unloaded cycling. MCAv and PCAv demonstrated a rapid increase followed by a return towards the initial baseline values during each sprint. The increase during the first sprint (MCAv: +31.9% ± 19.9%, PCAv: +32.5% ± 30.6%) was always greater than subsequent sprints (*p* ≤ 0.039). Each sprint was followed by a transient increase in MCAv and PCAv, which mirrored P_ET_CO_2_. SIE was followed by 60 min of hypocapnia, and blood pressure was significantly lowered 10–20 and 40–55 min post SIE. However, resting MCAv was not altered, and PCAv was only lower 5 min post SIE. Resting MCAv phase and gain, and PCAv phase were largely unaltered apart from the first 5 min post SIE. The ability to buffer changes in blood pressure during repeated squat‐stand maneuvers remained intact 60 min post SIE, although a greater fall in MCAv and PCAv was noted during a single sit‐to‐stand transition at this time.

## 1 INTRODUCTION

Evidence indicates that high‐intensity interval exercise can provide superior, acute improvements in peripheral vascular function compared to moderate‐intensity exercise (Weston, Koep, et al., 2022) and this may be at least partly explained by exposure to a greater shear stress stimulus (Green et al., 2002). Recent experimental data have also confirmed the importance of exercise‐induced shear as a stimulus for acute alterations in cerebrovascular function (Sakamoto et al., 2024). There is therefore value in understanding the cerebrovascular responses to different types of exercise. It is also particularly important to understand the intracranial responses to higher exercise intensities, which may provide more of a profound regulatory challenge to the brain (Calverley et al., 2020).

Sprint interval exercise (SIE) is a more demanding subtype of high‐intensity interval exercise, but one which has also shown to be a potent stimulus for peripheral vascular adaptation (Rakobowchuk et al., 2008). SIE provides an extreme cardiopulmonary challenge for the body, and regulatory mechanisms must be in place to protect against cerebral hemorrhage during times of maximal cardiac output. Interestingly, the few data which are available demonstrate that the blood velocity in the middle (MCAv) and posterior (PCAv) cerebral arteries (as a proxy of intracranial perfusion) peaks approximately 10 s into an “all‐out” sprint and then declines, but this is then followed by a new zenith during recovery (a “rebound” effect) (Curtelin et al., 2018; Labrecque et al., 2020). This suggests that brain blood flow is being actively dampened during maximal intensity exercise, but this regulation is either lost or altered during recovery. This has potential implications for repeated SIE. We are only aware of one study investigating the MCAv response to SIE involving repeated intervals (Weaver et al., 2020). The authors reported that MCAv significantly increased from baseline in response to the first sprint bout, but a dampened MCAv response to each subsequent sprint was observed. All bouts showed a “rebound” in MCAv at the onset of the recovery periods. However, we are not aware of any study which has documented the PCAv responses to repeated SIE, which is important given the regional differences previously observed at higher exercise intensities (Smith & Ainslie, 2017).

A return to pre‐exercise MCAv and PCAv values in the aftermath of SIE also represents a demanding regulatory challenge for the cerebrovasculature. Profound elevations in minute ventilation at the end of exercise increase the excretion of carbon dioxide (a fall in end‐tidal carbon dioxide; P_ET_CO_2_). As a leading determinant of brain blood flow (Smith & Ainslie, 2017; Steventon et al., 2018), this drives down MCAv and PCAv, at least in adults (Weston et al., 2021). Arterial carbon dioxide pressure then remains lower than normal post exercise during a period of bicarbonate replenishment and lactate metabolism required for acid: base regulation (Stringer et al., 1992). Additionally, blood pressure can be lowered during this time, due to attenuations in total peripheral resistance (Rossow et al., 2010) and baroreflex sensitivity (Stuckey et al., 2012), and an increase in the release of various vasodilators (Halliwill, 2001). It is imperative that brain blood flow is protected and maintained during this period of simultaneous hypocapnia and hypotension.

It has previously been shown that the regulation of MCAv is unaffected during post‐exercise hypocapnia and hypotension following moderate‐intensity exercise (Willie et al., 2013). However, SIE provides far greater perturbations in cardiac output, P_ET_CO_2_, and blood pressure than moderate‐intensity exercise, and thus presents more of an “extreme” regulatory challenge. For example, data are available demonstrating an alteration in the perfusion‐pressure relationship (cerebral autoregulation) in the immediate aftermath of exhaustive (Bailey et al., 2011; Ogoh et al., 2005), but not moderate‐intensity (Ogoh, Dalsgaard, et al., 2007) or high‐intensity (Whitaker et al., 2024) exercise. Additionally, both the magnitude of the delayed post‐exercise hypotensive response and the mechanisms underlying this phenomenon are understood to be intensity dependent (Halliwill, 2001; Jones et al., 2021; Rossow et al., 2010). This might further challenge brain blood flow regulation, given that the cerebrovasculature is less able to buffer against decreases than increases in blood pressure (Brassard et al., 2017). Finally, our current understanding of the post‐exercise responses is mainly driven by MCAv data. Little is understood regarding the PCAv responses, which is required for a more thorough understanding.

The temporal relationship between blood pressure and MCAv/PCAv during the recovery from SIE has not been completely described. Data have recently been published which demonstrate that this relationship was unaltered 25 min after a single, 30 s all‐out sprint cycle (Weston et al., 2025). We are not aware of any study that has considered whether the post exercise hypotension typically experienced after repeated SIE presents more of a regulatory challenge to forced changes in blood pressure. In other words, does the ability to protect against a fall in blood pressure (when standing) become further challenged when resting blood pressure is lower. The purpose of this paper was to (1) describe the MCAv, PCAv and P_ET_CO_2_ responses to repeated all‐out cycling exercise, (2) characterize the changes in MCAv and PCAv, and their relationships with P_ET_CO_2_ and blood pressure during 1 h of post exercise recovery, and (3) examine whether the ability to regulate MCAv and PCAv during forced changes in blood pressure is altered 60 min post SIE, i.e., during a possible period of simultaneous hypocapnia and hypotension.

## 2 MATERIALS AND METHODS

Ethical approval was granted by the University of Exeter Sport and Health Sciences Ethics Committee (ID: 845625). All participants provided written informed consent prior to participation in the study. The study was completed in accordance with the Declaration of Helsinki, apart from registration in a database.

### 2.1 Participants

Fourteen healthy, young adults (7 male, 7 female, age: 25.7 ± 4.9 y [mean ± SD], stature: 1.73 ± 0.10 m, body mass: 72.4 ± 11.2 kg, body mass index: 24.2 ± 2.5 kg/m^2^) were recruited for this study. Subjects were required to be between the ages of 18 and 39 years, and the exclusion criteria included any contraindication to exercise, recent concussion (within the last 3 months), or the use of medication known to influence blood pressure or vascular function. Participants were required to abstain from exercise and caffeine on the day of testing and to be at least 2 h postprandial.

### 2.2 Experimental protocol

**FIGURE 1:**
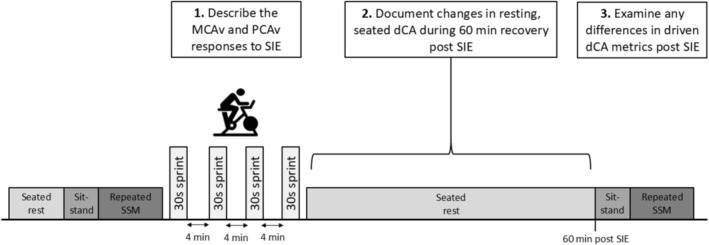
A schematic of the study protocol and aims. dCA, dynamic cerebral autoregulation; MCAv, middle cerebral artery blood velocity; PCAv, posterior cerebral artery blood velocity; SIE, sprint interval exercise; SSM, squat‐stand maneuvers.

Data were captured during a single experimental visit to the cerebrovascular laboratory at the University of Exeter (Figure 1). Participants visited the laboratory in the afternoon (∼13:30 ± 1 h) in order to account for any possible circadian effect on our outcomes of interest (Webb et al., 2024), and because the post exercise hypotensive response may be greater when exercise is performed in the afternoon (Jones et al., 2008). Thus, we attempted to maximize the autoregulatory challenge.

Following instrumentation, participants rested in the seated position for the assessment of baseline haemodynamic variables and spontaneous dynamic cerebral autoregulation (dCA), in line with the prevailing guidelines (Panerai et al., 2023). Upon completion of this resting period, participants were instructed to perform a single sit‐to‐stand transition for the assessment of driven dCA. This challenge was used as it replicates the phenomenon of postural hypotension and provided an opportunity to determine the occurrence of orthostatic hypotension, defined as a decrease in systolic blood pressure (SBP) of ≥ 40 mmHg and/or a reduction in diastolic blood pressure (DBP) of ≥ 20 mmHg during the first 15 s of standing (Freeman et al., 2011). Participants then remained standing until the achievement of a new physiological steady state, prior to performing repeated squat‐stand maneuvers (SSM) at a frequency of 0.05 Hz (10 s duty cycles) for 5 min (Smirl et al., 2015). This further assessment of dCA was included to facilitate comparisons with other data (Burma et al., 2020), given that dCA metrics may be protocol specific (Tzeng et al., 2012). The 0.05 Hz frequency was chosen as this may reflect myogenic mechanisms of cerebral autoregulation (Hamner & Tan, 2014).

Upon completion of all baseline measures, participants performed the SIE, before immediately returning to seated rest for 60 min. As described in Figure 1, data collection continued throughout this recovery period in order to examine spontaneous dCA post SIE. At 60 min, participants then repeated the single sit‐to‐stand transition and SSM.

### 2.3 Instrumentation

MCAv and PCAv were assessed using two 2 MHz transcranial Doppler (TCD) ultrasound probes (DWL, Compumedics, Germany), in accordance with standardized procedures (Smirl et al., 2016; Willie et al., 2011), and secured in place using an adjustable headset (DiaMon, DWL, Germany). MCAv and PCAv were measured on the right and left sides respectively, with two participants requiring the opposite due to difficulty in scanning. Beat‐to‐beat mean arterial blood pressure (MAP) was continuously measured by finger photoplethysmography with height correction (NIBP, ADInstruments, Colorado Springs, USA). Participants also had this hand supported in a sling with the finger aligned to heart level for all dCA assessments, in order to further account for any differences in hydrostatic pressure between the index finger and heart. This waveform was used to estimate cardiac output during the seated 60 min post‐exercise period via a three‐element Windkessel model, using the non‐invasive cardiac output software extension for LabChart Pro (ADInstruments, Colorado Springs, USA) which incorporates age and sex. Blood pressure was also measured using a brachial cuff (Dynamap Carescape, GE Healthcare, USA) before and at 5 min intervals post exercise. Systemic vascular conductance was calculated as cardiac output divided by MAP. Breath‐by‐breath expired gas was measured using a leak‐free facemask (Hans‐Rudolph, KS) connected to a ML206 Gas Analyzer (ADInstruments), apart from during exercise when P_ET_CO_2_ was assessed by a separate metabolic cart via a preVent flow sensor (Medgraphics Cardiorespiratory Diagnostics, UK) and time‐aligned to the MCAv and PCAv data. Both devices were always calibrated prior to use. All data were integrated (LabChart Pro) using an analogue to digital convertor at a sample rate of 1000 Hz (Powerlab 16/35, ADInstruments), apart from gas exchange data during the exercise which was exported breath‐by‐breath and then interpolated to 1 s averages and time aligned.

### 2.4 Spontaneous dCA

The relationships between resting MCAv/PCAv and blood pressure waveforms were assessed via transfer function analysis in order to generate dCA metrics of coherence, phase, and gain. In line with current recommendations, we present both absolute (cm s^−1^/mmHg) and normalized (%/%) gain (Panerai et al., 2023). These outcomes were determined from the seated rest immediately prior to SIE and in 5 min time bins immediately following exercise. This duration was used in an attempt to provide the best resolution of data to describe the recovery from SIE, whilst being in accordance with the minimum length of time to appropriately assess spontaneous dCA in the very low (0.02–0.07 Hz; VLF) and low (0.07–0.20 Hz; LF), as well as high (0.20–0.50 Hz; HF) frequency bands (Panerai et al., 2023). We conformed to these prevailing guidelines to derive metrics of spontaneous dCA across these frequency bands using our own software, which allowed for close scrutiny and detection of beat‐to‐beat MCAv/PCAv and blood pressure waveforms. This included Fourier transformation utilizing Welch's method, 100 s Hanning windows with 50% overlap and a critical coherence threshold of 0.34 (Panerai et al., 2023). In order to account for known data loss in the VLF band (Whitaker et al., 2024) caused by non‐linearity between input and output (Panerai et al., 2006), data which violated the minimum coherence threshold were re‐examined using 75% superposition. When phase wrap‐around was detected, data from those specific frequencies were removed from the parent frequency band averages.

### 2.5 Sit‐to‐stand challenge

We replicated previous approaches in this field (Labrecque et al., 2019; Whitaker et al., 2024). Specifically, participants sat with both feet flat on the floor and remained undisturbed to ensure an appropriate physiological baseline was captured. Baseline MCAv, PCAv, MAP, and P_ET_CO_2_ were defined as the average of the final 20 s prior to quickly transitioning to a standing position (<2 s) upon being prompted. Outcomes were continuously sampled for 60 s post standing, and the magnitude of the change in MCAv, PCAv, and MAP was calculated as the difference between baseline and their respective nadirs. These data were expressed in both absolute terms and as a relative percentage of the seated baseline. Cerebrovascular conductance index (CVCi) was calculated as MCAv or PCAv divided by MAP. The onset of the regulatory response was defined as the time when CVCi started to continuously increase following the post‐stand nadir, and this was agreed between two researchers (ES and BB). The rate of regulation (ROR) was calculated in order to consider the efficiency of the regulatory response during the first phase (1–7 s) of the hypotensive response, which is known to be uninfluenced by the arterial baroreflex (Aaslid et al., 1989; Ogoh et al., 2008). ROR was determined as the slope of CVCi between the time of the onset of the regulatory response and 7 s post standing normalized to the change in MAP (Whitaker et al., 2024).

### 2.6 Squat‐stand maneuvers

Participants started in a standing position, before being prompted to achieve a ~90° knee bend (“squat”) and then return to standing. Each squat and stand position was held for 10 s (i.e. 0.05 Hz). This approach ensures oscillations in MAP are within the high‐pass buffering range (i.e., <0.20 Hz), improves the signal‐to‐noise ratio of the dCA metrics and their reliability (Smirl et al., 2015), and is line with previous work concerned with dCA post sprint exercise (Weston et al., 2025). Participants were instructed to maintain normal ventilation throughout (i.e., avoid performing the Valsalva maneuver). The relationships between the MCAv/PCAv and blood pressure waveforms were examined via transfer function analysis, in line with current recommendations (Panerai et al., 2023) using commercially available software (Ensemble, Elucimed, Wellington, New Zealand). Coherence, phase, absolute and normalized gain were calculated at the point estimate for the driven frequency (0.05 Hz). There was no evidence of phase wrap‐around.

### 2.7 Sprint interval exercise

We replicated a standard SIE protocol (Burgomaster et al., 2008; Rakobowchuk et al., 2008). Following a 2 min unloaded warm up, participants completed four 30 s all‐out sprints against a resistance equating to 7.5% body mass. Participants accelerated maximally and attempted to maintain maximal speed throughout the 30 s sprint bouts. Participants remained seated on the cycle ergometer during the sprints and strong verbal encouragement was provided throughout each bout. Each sprint was followed by 4 min of unloaded pedaling recovery.

Mean MCAv and PCAv data were exported as 1 s averages and breath‐by‐breath P_ET_CO_2_ data were linearly interpolated to 1 s. All data were then time aligned to exercise start. Average MCAv, PCAv, and P_ET_CO_2_ data were calculated during each 30 s sprint and 4 min recovery period. Peak MCAv, PCAv, and P_ET_CO_2_ were determined as the highest 1 s data point achieved within each sprint and recovery. All outcomes were expressed in absolute values and as a percentage change from the last 30 s of the warm up (“baseline”) (%Δ).

### 2.8 Statistical analyses

Statistical analyses were performed using SPSS Statistics, version 28 (IBM) with statistical significance established when *p* < 0.05. All data are expressed as mean ± standard deviation (SD) unless otherwise stated. Prior to all analyses, a mixed model analysis of variance (ANOVA) was conducted to explore any main and interaction effect of sex. There were no significant main or interaction effects of sex for any outcome, so males and females were always grouped for subsequent analysis. A two‐way repeated measures ANOVA explored the main effects of vessel (MCAv and PCAv), time point (baseline, four sprint bouts and four recovery periods) and their interaction during the SIE for each variable (mean, peak, %Δ mean and %Δ peak). One‐way repeated measures ANOVA explored the main effect of time point (as before) for P_ET_CO_2_ variables during SIE. Pearsons correlation was used to explore relationships between power output, P_ET_CO_2_ and MCAv/PCAv during each 30 s sprint. These relationships were also explored during recovery; from the time of sprint cessation until peak MCAv/PCAv (~40 s), and from peak MCAv/PCAv until the end of the 4 min recovery period. A one‐way repeated measures ANOVA was also used to consider peak and mean power output during the four 30 s all‐out sprints, and the haemodynamic changes during recovery from SIE (MCAv, PCAv, P_ET_CO_2_ and MAP), with main effects for time (pre, and every 5 min post SIE). If Mauchley's assumption of sphericity was violated, the Greenhouse–Geisser correction was applied. Post hoc analyses using pairwise comparisons were performed using the LSD correction in the presence of significant main effects (Perneger, 1998). In order to be robust against incomplete cases, indices of spontaneous dCA during the 60 min recovery post SIE were compared to pre‐exercise values using a linear mixed model with time (each 5 min time bin) as fixed effects. Differences in driven dCA metrics from the sit‐to‐stand and SSM challenges pre and post SIE were assessed using paired samples *t*‐tests.

## 3 RESULTS

### 3.1 Cerebrovascular responses during sprint interval exercise

**FIGURE 2:**
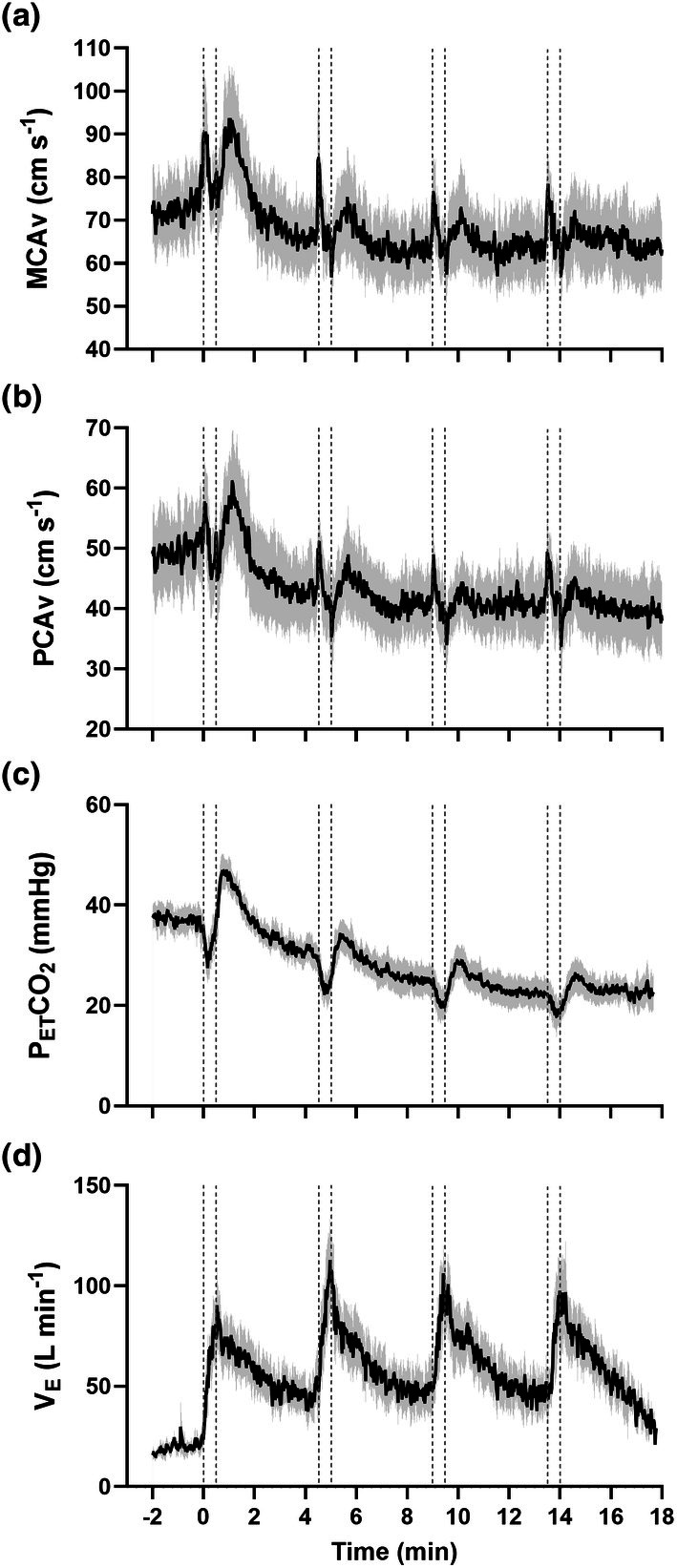
Physiological responses to four 30 s sprint interval bouts (S1–S4) and associated 4 min recovery periods. MCAv, middle cerebral artery blood velocity (a); PCAv, posterior cerebral artery blood velocity (b); P_ET_CO_2_, end‐tidal partial pressure of carbon dioxide (c); V_E_, minute ventilation (d). Data are represented as means ±95% confidence intervals. Due to data loss, *n* = 10 for MCAv and PCAv data, and *n* = 9 for P_ET_CO_2_ data.

Due to some signal loss during SIE, MCAv and PCAv data are presented for *n* = 10, and P_ET_CO_2_ are presented for *n* = 9 following equipment failure. Mean and peak power significantly declined during each of the four 30 s all‐out sprints (main effect of time *p* < 0.001 for both). Temporal MCAv, PCAv and P_ET_CO_2_ responses to the sprint interval exercise protocol are presented in Table 1 and visualized in Figure 2. Mean MCAv and PCAv responses were always greatest during sprint 1, with no differences between sprints 2, 3 and 4 (main effect of time *p* = 0.014 and *p* ≤ 0.001, respectively; post hoc pairwise comparisons are presented in Table 1). MCAv and PCAv were strongly and positively associated with P_ET_CO_2_ during sprints 2, 3 and 4 (*r* > 0.832, *p* < 0.001 for all), but not sprint 1 (*r* = −0.279, *p* = 0.136 and *r* = −0.152, *p* = 0.423). MCAv and PCAv were always significantly associated with power output during each sprint (*r* > 0.695, *p* < 0.001 for all).

**TABLE 1:** MCAv, PCAv, and P_ET_CO_2_ responses to repeated sprint intervals in healthy, young adults.

	Baseline	Sprint bout 1		Recovery 1		Sprint bout 2		Recovery 2		Sprint bout 3		Recovery 3		Sprint bout 4		Recovery 4	
	Mean	Mean	Peak	Mean	Peak	Mean	Peak	Mean	Peak	Mean	Peak	Mean	Peak	Mean	Peak	Mean	Peak
MCAv (cm s^−1^)	73.8 ± 12.5	81.7 ± 11.2^b^	73.8 ± 14.3^c^	101.4 ± 19.3^a,c^	90.4 ± 25.3	65.1 ± 13.1^a,h^	83.3 ± 19.3^a,h^	67.4 ± 11.1^d^	84.5 ± 13.7^a,d^	64.6 ± 12.9^a,h^	82.7 ± 16.0^a,h^	68.7 ± 10.3^d^	84.6 ± 12.5^a,d^	65.1 ± 13.4^a,h^	81.7 ± 15.8^a,h^
MCAv (%Δ)		13.9 ± 29.8^e,f^	31.9 ± 19.9^a,f,g^	−0.1 ± 8.1^c^	37.3 ± 10.1^a,c^	−2.8 ± 21.2^d^	27.8 ± 57.1	−11.8 ± 9.9^a,h^	12.4 ± 14.8^a,h^	−7.7 ± 14.9^d^	16.8 ± 25.0^d^	−12.4 ± 9.5^a,h^	12.5 ± 14.2^a,h^	−5.7 ± 15.0	16.4 ± 19.9^a,d^	−11.6 ± 11.5^a,h^	11.1 ± 14.6^a,h^
PCAv (cm s^−1^)	50.2 ± 10.6	50.7 ± 9.6^b^	64.8 ± 11.1^a,b^	47.3 ± 11.0^a,c^	66.6 ± 14.7^a,c^	43.6 ± 9.5^a,d^	53.9 ± 10.0^d^	41.8 ± 8.7^a,h^	55.4 ± 11.0^a,h,j^	41.7 ± 8.2^a,d^	51.0 ± 7.8^d^	40.7 ± 7.9^a,h^	52.3 ± 7.4^h^	43.5 ± 8.9^a,d^	52.7 ± 10.1^d^	40.4 ± 9.2^a,h^	51.3 ± 9.8^h,i^
PCAv (%Δ)		2.4 ± 15.4^b^	32.5 ± 30.6^a,b^	−6.2 ± 4.9^a,c^	32.5 ± 5.4^a,c^	−12.1 ± 16.6^a,d^	9.4 ± 21.2^d^	−16.7 ± 6.6^a,h^	11.0 ± 12.7^a,h,j^	−15.5 ± 16.4^a,d^	4.3 ± 21.3^d^	−18.4 ± 6.9^a,h^	5.7 ± 11.0^h^	−12.3 ± 16.1^a,d^	6.6 ± 19.2^d^	−19.5 ± 8.9^a,h^	3.0 ± 11.7^h,i^
P_ET_CO_2_ (mmHg)	36.8 ± 3.9	32.1 ± 5.2^a,b^	40.3 ± 5.2^b^	36.0 ± 3.4^c^	52.1 ± 3.4^a,c^	24.8 ± 4.5^a,b^	30.0 ± 3.8^a,b^	27.8 ± 4.6^a,c^	37.4 ± 5.7^c^	21.6 ± 5.0^a,b^	26.3 ± 5.1^a,b^	24.1 ± 4.5	31.8 ± 5.1^a,c^	19.9 ± 4.7^a,b^	23.2 ± 5.1^a,b^	23.3 ± 4.4^a,c^	28.9 ± 5.3^a,c^
P_ET_CO_2_ (%Δ)		−12.4 ± 13.4^a,b^	10.3 ± 16.9^b^	−1.9 ± 8.0	42.6 ± 14.5^a,c^	−32.7 ± 10.3^a,b^	−18.2 ± 8.9^a,b^	−24.2 ± 11.4^a,c^	1.7 ± 12.7^c^	−41.4 ± 11.7^a,b^	−28.4 ± 12.0^a,b^	−34.2 ± 11.7^a,c^	−13.2 ± 14.3^a,c^	−46.0 ± 10.8^a,b^	−36.7 ± 12.6^a,b^	−36.4 ± 11.6^a,c^	−21.0 ± 14.6^a,c^

*Note*: Data are presented as means ± standard deviation; *n* = 10 for MCAv and PCAv data, *n* = 9 for P_ET_CO_2_ data, *p* ≤ 0.05. %Δ percentage change from baseline. Abbreviations: MCAv, middle cerebral artery blood velocity; PCAv, posterior cerebral artery blood velocity; P_ET_CO_2_, end‐tidal partial pressure of carbon dioxide. ^a^Significantly different from baseline, ^b^Significantly different from all other sprint bouts, ^c^Significantly different from all other recovery periods, ^d^Significantly different from sprint 1, ^e^Significantly different from sprint 2, ^f^Significantly different from sprint 3, ^g^Significantly different from sprint 4, ^h^Significantly different from recovery 1, ^i^Significantly different from recovery 2, ^j^Significantly different from recovery 4.

When expressed either as absolute mean or peak velocity, MCAv was always greater than PCAv (main effect *p* < 0.001) and always decreased over time (main effect for time *p* < 0.001), with no significant vessel by time interaction effect (*p* > 0.239). When assessed as a percentage change relative to the warm up (%Δ), mean MCAv was greater than mean PCAv (main effect *p* < 0.001) and both similarly decreased over time (main effect for time *p* = 0.003; interaction effect *p* = 0.427). In contrast, the %Δ for peak values was not different between MCAv and PCAv (main effect of vessel *p* = 0.091), and decreased similarly over time between vessels (main effect for time *p* = 0.003; interaction effect *p* = 0.404).

MCAv and PCAv demonstrated biphasic responses during recovery from each sprint bout, characterized by an increase from baseline to peak values (~40 s), followed by a decline towards baseline levels. At the onset of the recovery period, both vessels demonstrated a “rebound” pattern characterized by a further peak and return towards baseline levels; however, this never exceeded the peak MCAv and PCAv values observed during the preceding sprint bout (*p* ≥ 0.228 for all). Peak MCAv during all recovery periods was significantly greater compared to during the warm up (*p* ≤ 0.039), with the initial recovery demonstrating the highest absolute value (101.4 ± 19.3 cm s^−1^, *p* < 0.001). Peak PCAv was only significantly raised from baseline during recovery period one (66.6 ± 14.7 cm s^−1^, *p* < 0.001) and two (55.4 ± 11.0 cm s^−1^, *p* = 0.032). PCAv peak %Δ during recovery periods was significantly higher than baseline during recovery periods one (+32.5% ± 5.4%, *p* < 0.001) and two (+11.0% ± 12.7%, *p* < 0.023). Furthermore, recovery one was significantly higher than all subsequent recovery periods (*p* ≤ 0.001). The increase in MCAv and PCAv during the initial ~40 s of recovery was always significantly and strongly correlated with P_ET_CO_2_ (*r* ≥ 0.742, *p* < 0.001 for all). In contrast, the relationship between MCAv/PCAv and P_ET_CO_2_ for the remainder of each 4 min recovery interval became weaker after each sprint (MCAv and PCAv; sprint 1 *r* = 0.944, *p* < 0.001 and *r* = 0.945, *p* < 0.001; sprint 2 *r* = 0.771, *p* < 0.001 and *r* = 0.779, *p* < 0.001; sprint 3 *r* = 0.571, *p* < 0.001 and *r* = 0.283, *p* < 0.001; sprint 4 *r* = 0.133, *p* = 0.057 and *r* = 0.107, *p* = 0.126).

Over the course of the repeated sprint interval protocol, there was a diminished cerebrovascular response in both vessels; as described by the post hoc pairwise comparisons in Table 1. Mean MCAv during sprint bout one (81.7 ± 11.2 cm s^−1^) was significantly higher than bout two (70.1 ± 9.9 cm s^−1^, *p* < 0.001), bout three (67.4 ± 11.1 cm s^−1^, *p* = 0.010), and bout four (68.7 ± 10.3 cm s^−1^, *p* = 0.024). Mean PCAv exhibited a similar response; sprint bout one (50.7 ± 9.6 cm s^−1^) was significantly higher than bout two (43.6 ± 9.5 cm s^−1^, *p* < 0.001), three (41.7 ± 8.2 cm s^−1^, *p* < 0.001), and four (43.5 ± 8.9 cm s^−1^, *p* = 0.012).

Mean MCAv of recovery periods also significantly diminished, with recovery one (73.8 ± 14.3 cm s^−1^) significantly higher than all subsequent recovery periods (recovery two: 65.1 ± 13.1 cm ^−1^s, *p* < 0.001, recovery three: 64.6 ± 12.9 cm s^−1^, *p* < 0.001, recovery four: 65.1 ± 13.4 cm s^−1^, *p* < 0.001). Mean PCAv of recovery one (47.3 ± 11.0 cm s^−1^) was also significantly higher than all others (recovery two: 41.8 ± 8.7 cm s^−1^, *p* < 0.001, recovery three: 40.7 ± 7.9 cm s^−1^, *p* < 0.001, recovery four: 40.4 ± 9.2 cm s^−1^, *p* < 0.001).

The initial rise in MCAv and PCAv at the onset of each sprint was not accompanied by an increase in P_ET_CO_2_. Apart from this, P_ET_CO_2_ elicited a similar response to MCAv and PCAv; reducing at the start of the sprint bouts, followed by a peak shortly after the onset of the recovery periods. P_ET_CO_2_ was sequentially lower for each sprint bout and every recovery period.

### 3.2 Post exercise rest

Continuous monitoring of all haemodynamic data was not possible for 3 participants. Of these, instrumentation was temporarily removed for one participant who experienced a hypotensive episode (although short of outright syncope), where their brachial MAP decreased to 65 mmHg 20 min post SIE.

**FIGURE 3:**
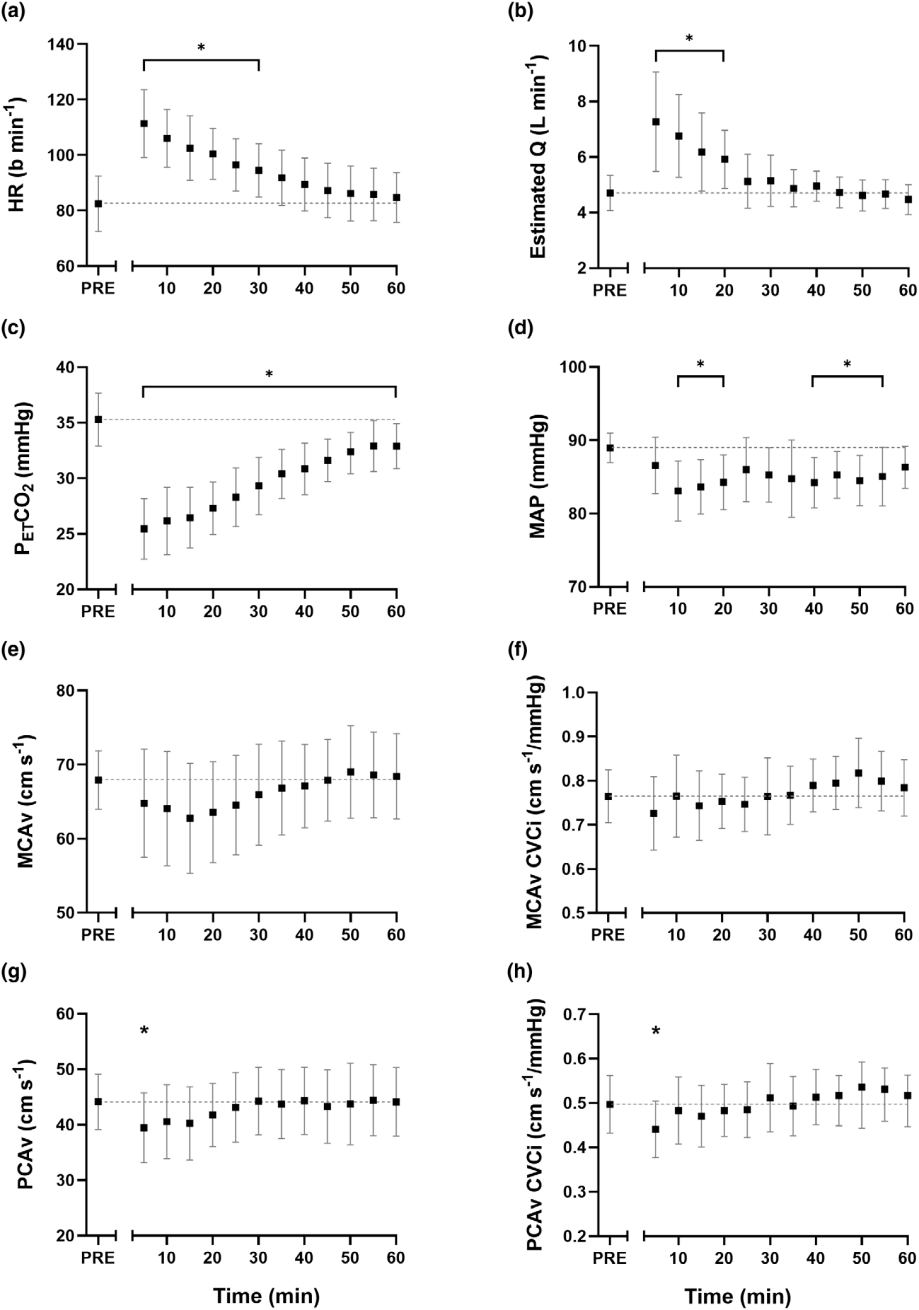
Haemodynamic variables measured during seated rest before (PRE) and every 5 min after the sprint interval exercise bout. HR, heart rate (a); Q, cardiac output (b); P_ET_CO_2_, end‐tidal partial pressure carbon dioxide (c); MAP, brachial derived mean arterial blood pressure (d); MCAv, blood velocity in the middle cerebral artery (e); CVCi, cerebrovascular conductance index (f, h); PCAv, blood velocity in the posterior cerebral artery (g). Data presented as means ±95% confidence intervals. Due to data loss in three participants, *n* = 11. * denote significant difference from pre exercise (please see text for *p* values).

Seated, resting haemodynamic variables recorded before and in the 60 min following SIE are presented in Figure 3. Heart rate and estimated cardiac output were elevated for the first 30 min (*p* < 0.034 for all) and 20 min (*p* < 0.042 for all) after SIE, respectively. P_ET_CO_2_ was always lower after SIE (*p* < 0.013 for all), but not by more than 10 mmHg at any point. MAP was also always lower than pre exercise values, although this did not achieve statistical significance at 5 min (*p* = 0.205), 25 min (*p* = 0.180), 30 min (*p* = 0.065), 35 min (*p* = 0.120) or 60 min post (*p* = 0.105). Despite this hypocapnia and hypotension, MCAv was never significantly different to pre exercise values (*p* > 0.158 for all), and PCAv was only different 5 min post SIE (*p* = 0.023). No differences in CVCi were observed for MCAv (*p* > 0.372 for all), and CVCi was only altered 5 min post SIE for PCAv (*p* = 0.019). In contrast, SVC was elevated during the first 20 min post SIE (*p* < 0.048 for all).

### 3.3 Spontaneous dCA

**FIGURE 4:**
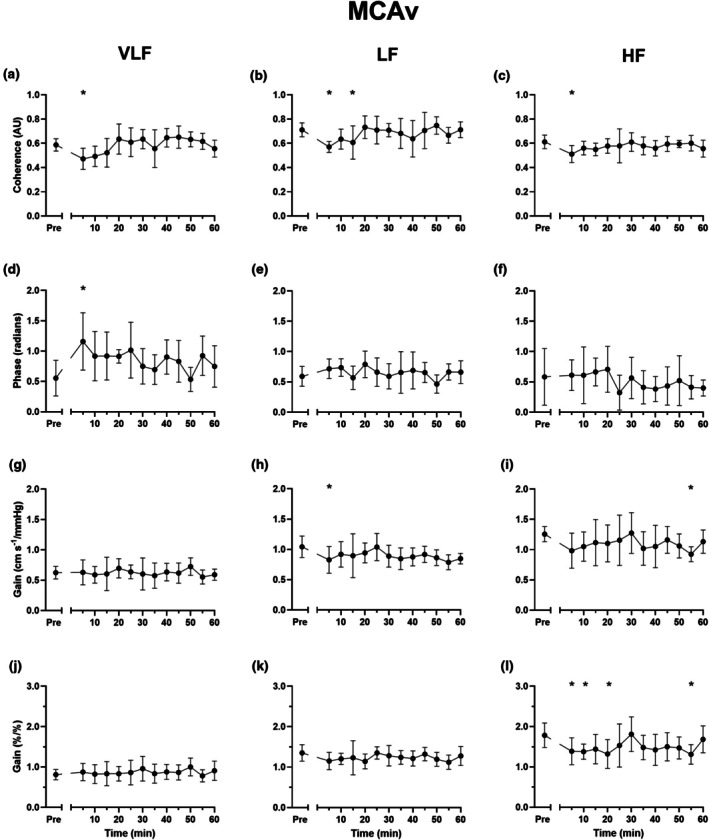
Spontaneous dynamic cerebral autoregulation indices of middle cerebral artery blood velocity (MCAv) during seated rest pre and post sprint interval exercise. Coherence (a–c), phase (d–f), absolute gain (g–i) and normalized gain (j–l). Data presented as means ±95% confidence intervals. VLF, very low frequency; LF, low frequency; HF, high frequency. * denote a statistically significant difference from pre exercise (please see text for *p* values).

**FIGURE 5:**
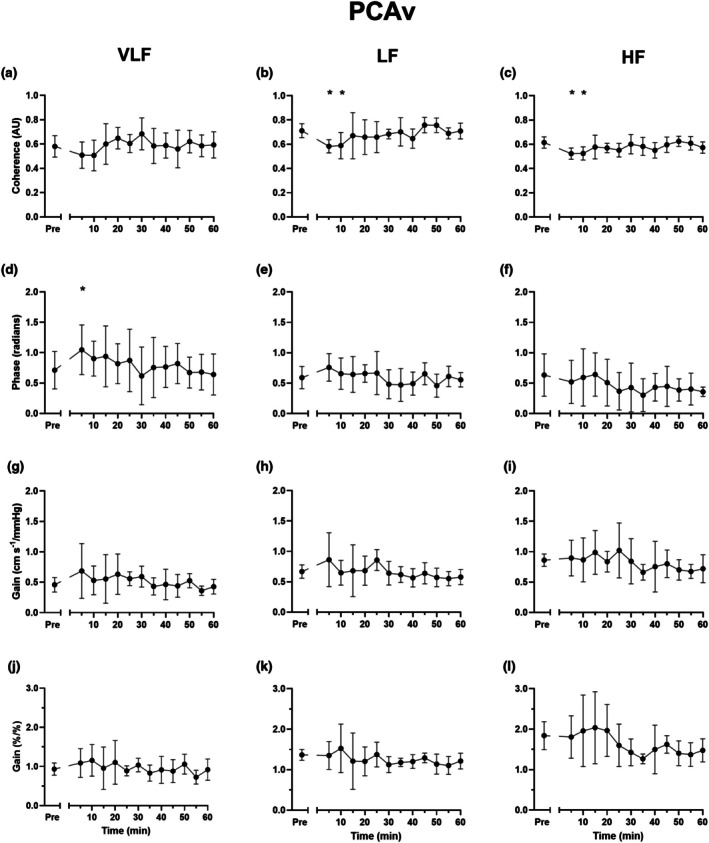
Spontaneous dynamic cerebral autoregulation indices of posterior cerebral artery blood velocity (PCAv) during seated rest pre and post sprint interval exercise. Coherence (a–c), phase (d–f), absolute gain (g–i) and normalized gain (j–l). Data presented as means ±95% confidence intervals. HF, high frequency; LF, low frequency; VLF, very low frequency. * denote a statistically significant difference from pre exercise (please see text for *p* values).

Indices of spontaneous dCA across the three frequency bands are presented in Figure 4 (MCAv) and Figure 5 (PCAv) and Table 2. Spontaneous dCA metrics for MCAv remained largely unchanged during the 60 min recovery period, with the exception of a decrease in coherence (*p* < 0.022 for all frequency bands), increase in VLF phase (*p* = 0.002), decrease in LF absolute gain (*p* = 0.042), and decrease in HF normalized gain (*p* = 0.037) 5 min post SIE. The decrease in HF normalized gain extended beyond 5 min and was significantly different to pre‐exercise values 10, 20, and 55 min post SIE (*p* < 0.037 for all). No such post exercise alterations in absolute or normalized gain were observed for PCAv across any frequency, although a similar increase in VLF phase was noted 5 min into the recovery period (*p* = 0.008).

**TABLE 2:** Power spectral densities for seated resting blood pressure and blood velocity pre and post sprint interval exercise.

	Pre	Post exercise											
		5 min	10 min	15 min	20 min	25 min	30 min	35 min	40 min	45 min	50 min	55 min	60 min
*VLF*													
BP (mmHg^2^)	384 ± 148	436 ± 340	537 ± 316	572 ± 336	565 ± 190	406 ± 138	475 ± 247	523 ± 254	512 ± 498	409 ± 270	324 ± 164	525 ± 503	471 ± 327
MCAv (cm s^2^)	270 ± 158	319 ± 204	352 ± 215	415 ± 199	**438 ± 223**	268 ± 116	257 ± 157	261 ± 187	290 ± 268	193 ± 97	254 ± 133	248 ± 191	240 ± 144
PCAv (cm s^2^)	197 ± 190	259 ± 229	351 ± 382	267 ± 188	238 ± 146	195 ± 48	258 ± 246	170 ± 125	169 ± 173	95 ± 56	119 ± 67	109 ± 68	123 ± 85
*LF*													
BP (mmHg^2^)	128 ± 89	183 ± 125	**252 ± 182**	**270 ± 248**	**267 ± 76**	196 ± 99	214 ± 81	**305 ± 154**	216 ± 135	205 ± 103	**237 ± 142**	**258 ± 130**	198 ± 77
MCAv (cm s^2^)	139 ± 83	169 ± 117	**265 ± 186**	190 ± 129	**249 ± 89**	227 ± 111	178 ± 74	**262 ± 145**	181 ± 98	167 ± 71	192 ± 103	200 ± 132	158 ± 80
PCAv (cm s^2^)	65 ± 51	114 ± 91	**132 ± 107**	105 ± 89	124 ± 64	**171 ± 86**	112 ± 74	**157 ± 144**	80 ± 62	81 ± 56	89 ± 56	97 ± 73	74 ± 60
*HF*													
BP (mmHg^2^)	12 ± 7	23 ± 25	23 ± 14	23 ± 7	23 ± 14	15 ± 12	15 ± 8	23 ± 16	19 ± 11	18 ± 8	21 ± 8	**29 ± 20**	19 ± 17
MCAv (cm s^2^)	27 ± 13	35 ± 36	49 ± 53	**60 ± 61**	41 ± 38	31 ± 20	37 ± 30	34 ± 21	32 ± 23	28 ± 20	34 ± 19	35 ± 28	38 ± 27
PCAv (cm s^2^)	12 ± 8	**34 ± 44**	20 ± 15	30 ± 23	24 ± 14	21 ± 15	17 ± 9	18 ± 14	18 ± 19	13 ± 10	17 ± 12	18 ± 18	17 ± 16

*Note*: Data are presented as mean ± standard deviation; *n* = 11. Data in bold are significantly different from pre‐exercise values.

Abbreviations: BP, blood pressure; HF, high frequency; LF, low frequency; MCAv, middle cerebral artery blood velocity; PCAv, posterior cerebral artery blood velocity; VLF, very low frequency.

### 3.4 Sit to stand challenge

**FIGURE 6:**
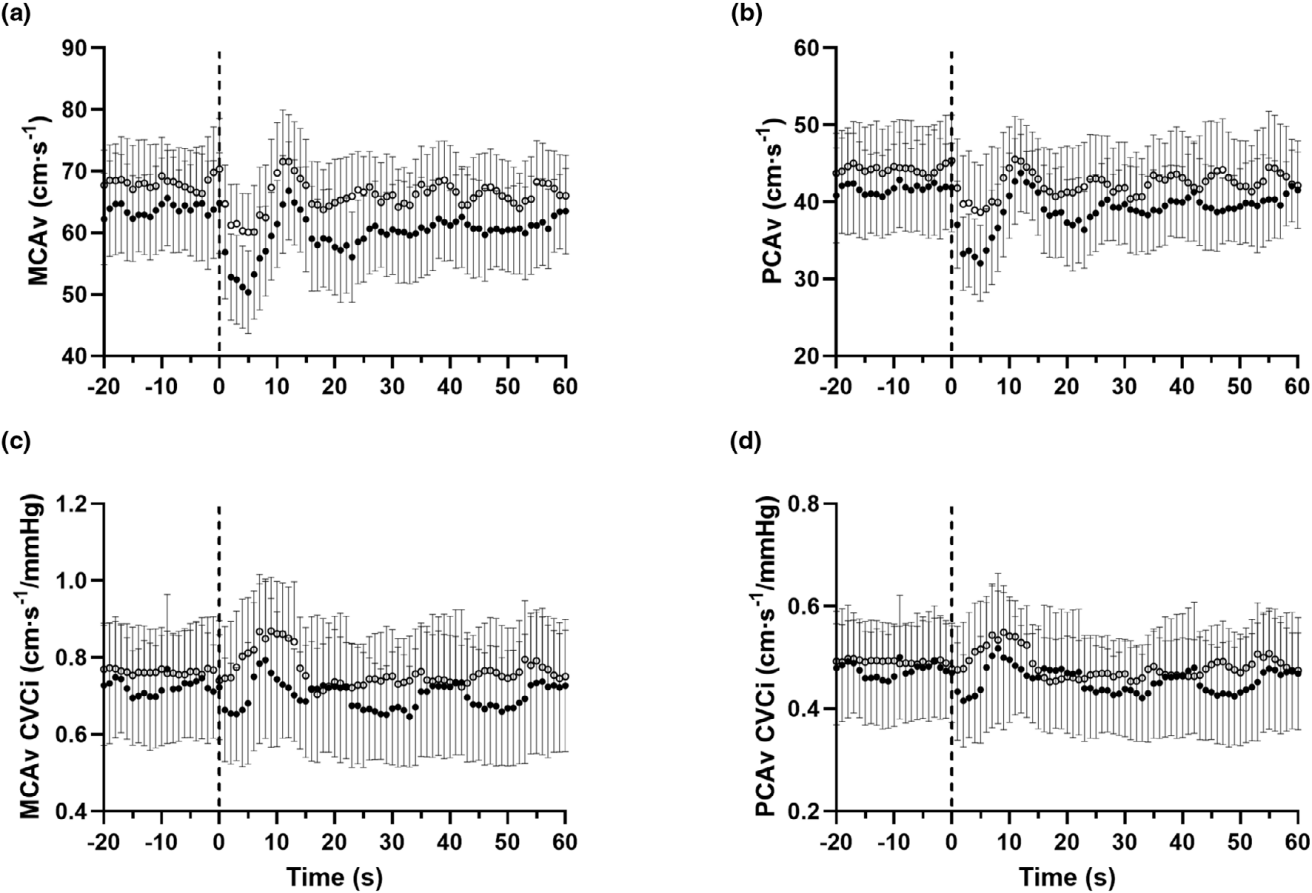
Cerebrovascular responses to a single sit to stand transition (as denoted by the dotted line) pre (○) and post (●) sprint interval exercise. Data presented as means ±95% confidence intervals. MCAv, blood velocity in the middle cerebral artery (a); PCAv, blood velocity in the posterior cerebral artery (b); CVCi, cerebrovascular conductance index (c, d).

The haemodynamic responses to the sit‐to‐stand challenge are presented in Figure 6. No participant experienced postural hypotension upon standing either pre or post SIE.

Transitioning from a sitting to standing position decreased MAP by similar amounts pre and 60 min post exercise (20.2 ± 9.8 vs. 21.7 ± 12.5 mmHg, *p* = 0.605). In contrast, the absolute fall in brain blood velocity was greater 60 min post exercise for MCAv (−12.7 ± 7.8 vs. −17.5 ± 5.6 cm s^−1^, *p* = 0.054) and PCAv (−9.2 ± 4.6 vs. −12.9 ± 6.5 cm s^−1^, *p* = 0.048). This fall was statistically significant when expressed as a percentage decrease from seated baseline for both MCAv (−18.4 ± 10.8 vs. −27.7% ± 6.6%, *p* = 0.006) and PCAv (−21.1 ± 10.7 vs. −31.2% ± 11.1%, *p* = 0.016). The onset of the regulatory response was unaltered post SIE for MCAv (4.5 ± 1.9 s vs. 4.7 ± 1.9 s, *p* = 0.683) and PCAv (4.3 ± 1.9 vs. 4.9 ± 1.8 s, *p* = 0.231). ROR was greater after exercise for MCAv (0.002 ± 0.001 vs. 0.005 ± 0.004 AU, *p* = 0.021), although this just failed to achieve statistical significance for PCAv (0.001 ± 0.001 vs. 0.003 ± 0.003 AU, *p* = 0.056).

### 3.5 Squat‐stand maneuvers

Key dCA outcomes from the SSM are presented in Table 3. Blood pressure power was significantly greater post SIE (*p* = 0.040), with no change in MCAv and PCAv power spectrum densities. No significant differences were observed post SIE for coherence, phase, absolute gain, or normalized gain at any point across the cardiac cycle for MCAv or PCAv.

**TABLE 3:** Power spectrum densities of blood pressure and cerebral blood velocity during repeated squat‐stand maneuvers at 0.05 Hz.

	Pre	Post	*p* value
*PSD*			
MAP (mmHg^2^)	31,740 ± 21,632	43,318 ± 34,219	**0.040**
MCAv (cm s^2^)	11,663 ± 9130	12,866 ± 11,288	0.377
PCAv (cm s^2^)	5343 ± 4597	7289 ± 7125	0.102
*MCAv*			
Coherence (AU)	0.91 ± 0.11	0.95 ± 0.05	0.176
Phase (radians)	0.69 ± 0.21	0.62 ± 0.30	0.278
Absolute gain (cm s^−1^/mmHg)	0.56 ± 0.15	0.54 ± 0.19	0.406
Normalized gain (%/%)	0.82 ± 0.21	0.91 ± 0.26	0.077
*PCAv*			
Coherence (AU)	0.91 ± 0.09	0.96 ± 0.02	0.085
Phase (radians)	0.68 ± 0.21	0.57 ± 0.26	0.070
Absolute gain (cm s^−1^/mmHg)	0.40 ± 0.14	0.41 ± 0.14	0.715
Normalized gain (%/%)	0.92 ± 0.32	1.02 ± 0.30	0.231

*Note*: Data are presented as means ± standard deviation; *n* = 11. Statistical significance highlighted in bold.

Abbreviations: MCAv, middle cerebral artery blood velocity; PCAv, posterior cerebral artery blood velocity; PSD, power spectral density.

## 4 DISCUSSION

This is the first study to describe the temporal MCAv, PCAv, and P_ET_CO_2_ responses to SIE and consider metrics of cerebral autoregulation during 60 min of seated recovery post SIE. We observed that MCAv and PCAv elicit similarly patterned responses to SIE: a biphasic response to the sprint bouts and a “rebound” at the onset of the recovery periods. The latter was closely mirrored by the P_ET_CO_2_ response. We also observed that the MCAv and PCAv responses were greatest in response to the first sprint interval. MCAv and PCAv remained largely unaltered during the 60 min immediately following SIE, despite hypocapnia and hypotension. Similarly, indices of spontaneous dCA remained largely stable during this recovery period, apart from some disturbance to the pressure‐perfusion relationship mostly during the first 5 min. Finally, transitioning to a standing position elicited a greater fall in MCAv and PCAv, despite similar changes in blood pressure, 1 h after exercise. However, no changes in driven dCA metrics were observed at this time during repeated squat‐stand transitions. Collectively, these data describe the extent of the haemodynamic challenge experienced during and after SIE, and highlight that MCAv and PCAv are predominantly protected from the associated hypocapnic and hypotensive period experienced thereafter.

### 4.1 Physiological responses to SIE

The biphasic MCAv and PCAv responses observed in this study align with previous findings (Curtelin et al., 2018; Labrecque et al., 2020), although we observed greater percent changes from baseline. This is likely due to methodological differences, including higher sprint intensity and unrestricted cadence compared to prior protocols. Participant characteristics (e.g., trained females vs. mixed cohort) and the transition from warm‐up into the sprints may have further contributed (Labrecque et al., 2020). However, the results support previous findings that cerebral blood velocities dramatically increase at the onset of high intensity exercise and then fall (Curtelin et al., 2018; Labrecque et al., 2020). Notably, this study extends previous work by examining regional responses across repeated sprint bouts offering a more representative model of high‐intensity exercise. We also observed that the relationship between P_ET_CO_2_ and MCAv/PCAv was only present in the latter 3 sprints, as P_ET_CO_2_ declined during the initial sprint effort – which has been reported elsewhere (Curtelin et al., 2018). In contrast, MCAv and PCAv were always strongly associated with power output during each sprint. Thus, the increase in MCAv and PCAv during sprint 1 might be more related to the initial increase in MAP at the onset of maximal exertion.

The “rebound” MCAv and PCAv response during recovery periods (Figure 2a,b) is also in agreement with previous findings (Labrecque et al., 2020; Weaver et al., 2020). A unique aspect of SIE is the rapid drop in blood pressure following sprint cessation. Specifically, a 26% reduction in MAP has been observed within 3 s post sprint (Willie et al., 2014). However, no such immediate fall in MCAv/PCAv was observed in our study. We attempted to collect beat‐to‐beat MAP data during the exercise in order to provide some regulatory insight, but were unable to secure reliable data. However, and in accordance with Labrecque et al., significant MCAv and PCAv peaks followed the initial sprint, coinciding and significantly correlating with elevated P_ET_CO_2_ (Labrecque et al., 2020), which is in keeping with its known regulatory role (Smith & Ainslie, 2017; Steventon et al., 2018). Subsequent recovery periods elicited significant MCAv peaks relative to baseline, but our work extends the field by including PCAv responses, and these demonstrated significant increases in the first two recovery periods only. Whilst the middle and posterior cerebral arteries perfuse different regions of the brain, it is possible that the blunted reactivity to changes in arterial carbon dioxide concentration of the vertebral arteries which supply the PCA may explain the attenuated PCAv response in later recovery periods (Sato et al., 2012).

MCAv and PCAv responses to the SIE protocol diminished over time, with the initial sprint and recovery periods significantly higher than subsequent bouts, aligning with previous research (Weaver et al., 2020; Weston et al., 1985). This dampened response may be related to exercise‐induced reductions in P_ET_CO_2_, consistent with hyperventilatory hypocapnic cerebral vasoconstriction caused by high‐intensity exercise (Lucas et al., 2015; Ogoh & Ainslie, 2009; Whitaker et al., 2022); a neuroprotective mechanism employed by the brain to modulate against cerebral hyperperfusion (Curtelin et al., 2018; Lucas et al., 2015). Furthermore, the reductions to both MCAv and PCAv – predominantly supplied by the internal carotid arteries and vertebral arteries, respectively – suggest a coordinated neuroprotective response to regulate cerebral perfusion (Lucas et al., 2015; Sato et al., 2011). Duplex sonography to determine changes in volumetric flow to the brain would further this insight, although it may be prohibitively challenging to perform. Additionally, continuous assessment of MAP (whilst challenging during SIE) would add important insight here. It has previously been shown that the MAP increase during SIE is linearly related to power output (Curtelin et al., 2018), which declined over the 4 repeated 30 s sprint bouts. This could plausibly contribute to our observations of a diminishing increase in MCAv and PCAv with each sprint interval.

### 4.2 Regulation of MCAv and PCAv during recovery from SIE

Despite substantial alterations during SIE, MCAv and PCAv returned to pre‐exercise values within 5 and 10 min, respectively, and then remained unchanged for the remainder of the 60 min observation period (Figure 3e,g). We find this to be striking, given the 20 min elevation in estimated cardiac output and SVC, and sustained hypocapnia and hypotension experienced during this 60 min period. Such findings are in line with, and extend, the observations after moderate intensity exercise (Willie et al., 2013). These authors demonstrated that MCAv returned to pre‐exercise values within 10 min, despite hypocapnia (approximately −3 mmHg) and a ~10 mmHg reduction in MAP. This quick return to near‐baseline values in MCAv/PCAv despite these perturbations presumably speaks to the strong homeostatic drive and complex safeguards in place to maintain appropriate cerebrovascular perfusion, which differ from the prolonged post‐exercise hyperaemia experienced by the active limb (Bangsbo et al., 1991). Indeed, metrics of spontaneous dCA across different frequency bands for both MCAv and PCAv regulation were largely unchanged during this period (apart from some initial small disturbances), which again tallies with observations made in the 1 h following moderate intensity exercise (Willie et al., 2013) and 10 min post heavy intensity exercise (semi‐recumbent cycling at ~150 b min^−1^) (Ogoh, Fisher, et al., 2007). Interestingly, it has been shown that a single bout of high‐intensity interval exercise may lower MCAv VLF gain in healthy, older adults, although this only achieved statistical significance 30 min post, rather than immediately afterwards (Whitaker et al., 2024). We did not observe such alterations, and suspect this may be at least partly due to population differences (60 ± 13 y vs. 26 ± 5 y, and 28.9 ± 6.6 kg/m^2^ vs. 24.2 ± 2.5 kg/m^2^).

Our findings that some alterations in the perfusion‐pressure relationship can occur during early recovery (~5 min) from maximal intensity exercise are consistent with previous observations immediately after exhaustive resistance (Koch et al., 2005) and cycling (Bailey et al., 2011) exercise, albeit with some nuanced differences. For example, Koch and colleagues also observed a reduction in MCAv LF gain during early (90 s) recovery, but not the increase in phase we report here. However, multiple differences between these studies (the type of exercise, recovery timeline, manner of autoregulation assessment) preclude direct comparison. For example, Bailey et al. used the thigh cuff technique to elicit a transient drop in MAP (Bailey et al., 2011), which reflects a driven autoregulatory challenge. Despite this, these data indicate that post‐exercise alterations in the perfusion‐pressure relationship may occur provided that exercise is of a sufficient intensity, but these may be short‐lived. For example, our own group has observed an increase in dCA gain during SSM 10 min, but not 45 min, after 4 sets of weighted squats (Smail et al., 2023).

We did not observe any differences in dCA metrics during the SSM 60 min after SIE. We cannot rule out that this might be related to the timing of this single assessment (Smail et al., 2023), but we note that Weston et al. recently reported no changes in phase, gain or coherence during SSM 25 min post a single 30 s all‐out cycling bout (Weston et al., 2025). However we noted a greater fall in MCAv and PCAv during the single sit‐to‐stand test (i.e., a worsened ability to buffer this orthostatic challenge, Figure 5a,b), although the magnitude of this difference in MCAv/PCAv change and onset of regulation was small – particularly for PCAv. This seeming contradiction between dCA assessments has been observed before – Labrecque et al. demonstrated sex differences in the response to a sit‐to‐stand test which were not apparent using the 0.05 Hz SSM challenge (Labrecque et al., 2019). This likely reflects the quandary of quantification for the field (Brassard et al., 2023), which is why a multi‐modal assessment of autoregulation is advocated. From a translational perspective, the sit‐to‐stand test has clear application regarding orthostatic intolerance, which has been shown to be exaggerated post exercise due to hypocapnia, hypotension, lower venous return and baroreflex unloading (Lucas et al., 2008). We note that the response to a sit‐to‐stand test was largely unaltered 35 min after high‐intensity interval exercise in older adults (Whitaker et al., 2024). Again, differences in sample populations cloud comparisons (Murrell et al., 2009), but our finding of a more pressure‐passive response post SIE adds to tilt‐table data demonstrating a greater post exercise orthostatic intolerance with increasing exercise intensity, possibly due to greater falls in P_ET_CO_2_ (Mundel et al., 2015).

### 4.3 Study considerations

This study has some methodological strengths; the nature of the SIE protocol ensured that all participants were sprinting to maximal effort, providing commensurable results for comparison between participants and against potential future research. This was also the first study to describe the responses in both the MCAv and PCAv during SIE and in the aftermath of such exercise, and to consider these responses in light of key regulators of brain blood flow: P_ET_CO_2_ and MAP, including a multi‐modal assessment of cerebral autoregulation in line with prevailing guidelines (Panerai et al., 2023). However, this study is not without its limitations. Chiefly, we recognize the known variance in our outcomes of interest and our own small sample size. Thus, we caution against interpreting non‐statistically significant differences as evidence of no physiological change.

An important consideration is that MCAv and PCAv were measured using TCD, a surrogate measure of cerebral blood flow that relies on the assumption of diameter constancy (Ainslie & Hoiland, 2014). This assumption may be violated when P_ET_CO_2_ values differ by more than +9 mmHg or −13 mmHg from a stable resting baseline (Ainslie & Hoiland, 2014; Coverdale et al., 2014), and collated research has demonstrated that cerebral blood flow may be overestimated by up to 10% during hypocapnia of 15 mmHg P_ET_CO_2_ below baseline (Ainslie & Hoiland, 2014). The recorded P_ET_CO_2_ fell below this acceptable range for the assumption of diameter constancy during SIE (Figure 2c), but was not observed in the 60 min post exercise observation period. However, the effect of exercise on P_ET_CO_2_ versus controlled alterations of P_ET_CO_2_ at rest does not offer an appropriate comparison to fully understand vascular behavior during these different stimuli. Due to the many advantages of TCD, such as high temporal resolution, non‐invasive implementation, portability, and adaptability (Willie et al., 2011), it is considered the most suitable and practical method to estimate regional brain perfusion during exercise (Ainslie & Hoiland, 2014).

A further limitation of this study was the lack of beat‐to‐beat blood pressure measurement during the exercise bout. We attempted to do so, but were unable to establish reliable data using non‐invasive finger photoplethysmography, which is a recognized technological challenge (Labrecque et al., 2020). Exercise‐induced changes in mean arterial pressure are likely to be a contributory factor to regional cerebrovascular responses (Smith & Ainslie, 2017). For example, the change in MCAv has been shown to be strongly correlated with the change in MAP during repeated intervals of moderate intensity cycling (Klein et al., 2019). However, it is likely that the brain employs neuroprotective mechanisms to temper the cerebral blood flow response to increases in blood pressure during SIE (Curtelin et al., 2018). Therefore, the extent of the contribution of MAP to the time course of MCAv and PCAv responses during SIE in this study is unknown.

This study did not control for contraceptive use or menstrual phase, although we are not aware of any data highlighting that this might influence the cerebrovascular response to exercise (Weston, Barker, et al., 2022). However, resting CBV, measured using TCD and ultrasonography of carotid arteries, is affected by the menstrual cycle and associated hormones (Krejza et al., 2001; Peltonen et al., 2016). Additionally, sex, use of oral contraceptives, fitness, and training status have been shown to influence dCA (Abidi et al., 2017; Labrecque et al., 2019) and the mechanisms underlying post exercise hypotension (Senitko et al., 2002). We did not measure contraceptive use, fitness, or training status. We also did not observe any interaction effect of sex when considering pre vs. post exercise outcomes, but we recognize that our study was not powered to explore this. Therefore, our pooling of males and females is made with caution, and future work is needed to address this directly.

### 4.4 Conclusion

In conclusion, MCAv and PCAv elicited similar, biphasic responses to repeated sprint bouts and “rebounded” at the onset of recovery, which largely mirrored the P_ET_CO_2_ response. MCAv and PCAv diminished over the course of the protocol to values lower than baseline, alongside decreases in P_ET_CO_2_. MCAv and PCAv were largely unaltered during the 60 min recovery period, despite profound changes in cardiac output, hypocapnia and hypotension, and we observed only small disruptions to the perfusion‐pressure relationship during this time. Collectively, these data describe the haemodynamic challenge of SIE, and the impressive capacity to protect MCAv and PCAv in the aftermath, despite physiological stressors.

## AUTHOR CONTRIBUTIONS

**E. Scott:** Conceptualization; data curation; formal analysis; investigation; methodology; project administration. **E. Langson‐Justice:** Conceptualization; data curation; formal analysis; investigation; methodology; project administration. **O. Smail:** Data curation; formal analysis; investigation; methodology; software. **A. B. Lester:** Data curation; formal analysis; investigation; methodology. **R. Oliveira:** Conceptualization; formal analysis; investigation. **C. J. A. Pugh:** Conceptualization; formal analysis; investigation. **M. E. Weston:** Conceptualization; formal analysis; investigation. **B. O. Bond:** Conceptualization; data curation; formal analysis; investigation; methodology; project administration; resources; software; supervision; validation; visualization.

## FUNDING INFORMATION

This work was not supported by external funding.

## CONFLICT OF INTEREST STATEMENT

The authors have no conflicts of interest to disclose.

## Data Availability

The data that support the findings of this study are available from the corresponding author upon reasonable request. For the purpose of open access, the author has applied a Creative Commons Attribution (CC BY) license to any Author Accepted Manuscript version arising from this submission.
